# Small Cell Lung Cancer from Traditional to Innovative Therapeutics: Building a Comprehensive Network to Optimize Clinical and Translational Research

**DOI:** 10.3390/jcm9082433

**Published:** 2020-07-30

**Authors:** Shanmuga Subbiah, Arin Nam, Natasha Garg, Amita Behal, Prakash Kulkarni, Ravi Salgia

**Affiliations:** Department of Medical Oncology and Therapeutics Research, City of Hope National Medical Center, Duarte, CA 91010, USA; ssubbiah@coh.org (S.S.); anam@coh.org (A.N.); ngarg@coh.org (N.G.); abehal@coh.org (A.B.); pkulkarni@coh.org (P.K.)

**Keywords:** small cell lung cancer, translational research, immunotherapy, clinical trials, team medicine, community practice

## Abstract

Small cell lung cancer (SCLC) is an aggressive, complex disease with a distinct biology that contributes to its poor prognosis. Management of SCLC is still widely limited to chemotherapy and radiation therapy, and research recruitment still poses a considerable challenge. Here, we review the current standard of care for SCLC and advances made in utilizing immunotherapy. We also highlight research in the development of targeted therapies and emphasize the importance of a team-based approach to make clinical advances. Building an integrative network between an academic site and community practice sites optimizes biomarker and drug target discovery for managing and treating a difficult disease like SCLC.

## 1. Introduction

Lung cancer is the leading cause of cancer deaths in both men and women in the United States [1]. Small cell lung cancer (SCLC) is a subtype of lung cancer that has an incidence of 13% and is strongly associated with smoking [2,3]. A distinct biology, aggressive clinical course with distant metastasis, and poor survival outcomes characterize SCLC. The disease is classified into extensive stage and limited stage. While limited stage SCLC (LS-SCLC) is disease confined to one hemithorax that can be enclosed within a radiation field, extensive stage SCLC (ES-SCLC) is more prevalent (66%) and includes malignant pleural or pericardial effusions along with distant metastasis [4]. Despite the bleak prognosis, standard chemotherapies for patients with SCLC have not changed significantly in the last 30 years. However, recently, immunotherapy with checkpoint inhibitors have shown promising efficacy in advanced disease [5,6]. The lack of advances in SCLC therapies are partly due to disease complexity, research recruitment, and resource utilization. Thus, collaborative efforts between academic and community practices that combine knowledge, skills, experiences, and expertise of academicians, clinicians, and researchers, can accelerate advances in treatment and patient care. Academic centers are critical in the advancement of cancer treatment, but community hospital care plays an equally important and complementary role. Indeed, academic–community collaboration, or ‘team medicine’, has become an emerging culture to advance and shape clinical care.

In this article, we first highlight the current standard treatments in SCLC as well as recent advances in immunotherapies. We also review potential targeted therapies and underscore the importance of a team-based approach toward SCLC based on our experience at the City of Hope (COH).

## 2. Current Standard Therapies

### 2.1. Radiation Therapy

For LS-SCLC the standard of care is chemotherapy with concurrent radiation therapy [7]. Two meta-analyses established that concurrent cisplatin and etoposide treatment combined with radiation therapy improves survival compared to chemotherapy alone [8,9], although the dosage (once daily vs. twice daily) of radiation with chemotherapy remains equivocal. One study showed a significant survival advantage of patients who received 1.5 Gy in 30 fractions twice daily compared to 1.8 Gy in 25 fractions after a median follow-up of 8 years [10]. However, a more recent trial that randomized patients to receiving 1.5 Gy twice daily fractions (45 Gy dose) or 2 Gy once daily fractions (66 Gy dose) concurrently with platinum based chemotherapy showed that survival outcomes did not differ between the regimens, although the trial was not powered for equivalence [11]. The ongoing trial of CALGB 30,610 comparing 45 Gy twice daily to 70 Gy once daily (NCT00632853) is likely to shed more light on this issue.

### 2.2. Chemotherapy

Regardless of the stage, platinum with etoposide (EP) is the standard of care for patients with SCLC in the United States. Outside the United States, some patients are given platinum with irinotecan as an alternative treatment [12,13,14]. The overall response rates (ORR) range from 40–70% with up to 10% of the patients achieving complete radiographic response, and the median overall survival (OS) spans 7–12 months, with a two-year survival rate of less than 5% [15]. Eventually, however, most SCLC tumors become resistant to chemotherapy resulting in disease progression. For patients with disease relapse, topotecan as a single agent is the only approved second-line drug that has demonstrated increased survival compared to supportive care [16,17]. Nonetheless, the ORR of patients treated with topotecan is only about 5% [18] and even worse, in SCLC patients who develop disease recurrence within 3 months of the first-line platinum doublet chemotherapy, in which case topotecan is ineffective. Other chemotherapeutic agents such as gemcitabine, docetaxel, paclitaxel, temozolomide, irinotecan, and vinorelbine, may be used in certain cases based on limited clinical evidence [19,20,21,22,23,24]. Amrubicin is an anthracycline agent that has been developed more recently and approved only in Japan for second-line therapy [25]. Unfortunately, beyond second-line therapy, currently there are no standard guidelines of care although, newer immunotherapies appear promising in some patients.

### 2.3. Surgery

Compared to non-small cell lung cancer (NSCLC), SCLC is rarely treated surgically. However, over the years the fraction of SCLC patients treated surgically has increased considerably from 14.9% in 2004 to 28.5% in 2013. This is at least in part due to availability of better diagnostic tools in the form of positron emission tomography (PET) scans and increasing usage of low-dose computed tomography (CT) screening. Randomized trials reported in the late 1960s and early 1970s showed no survival advantage for surgery alone or in combination with radiation therapy compared with radiation therapy alone [26,27]. Subsequently, it was reported that chemotherapy given sequentially with radiation and then randomized to surgery vs. non-surgical group did not show any benefit to surgery [28]. However, recently there have been increasing numbers of retrospective studies showing survival benefit of surgery compared to non-surgical therapy [29,30,31,32]. For example, a meta-analysis published by Liu et al. [33], which included two randomized control trials described above and thirteen retrospective studies for a total of 41,483 patients, concluded that surgical resection significantly improved overall survival when compared to non-surgical treatment (hazard ratio (HR) = 0.56, *p* < 0.001) for retrospective studies, and in the two randomized trials there was no survival advantage to the surgical arm. This meta-analysis also showed that lobectomy was associated with superior OS compared with sub lobar resection (HR = 0.64, *p* < 0.001). Based on these data, the National Comprehensive Cancer Network (NCCN) also recommends surgery for T1-2N0M0 SCLC provided preoperative evaluation of mediastinal lymph nodes are done. Unfortunately, there are no ongoing randomized trials evaluating surgery in SCLC, since less than 5% of patients present with stage I SCLC. However, a collaborative engagement with community clinic sites where majority of cancer patients are seen and academic institutes similar to COH should help accrue enough patients to conduct a prospective trial.

## 3. Novel Therapies

**Figure 1:**
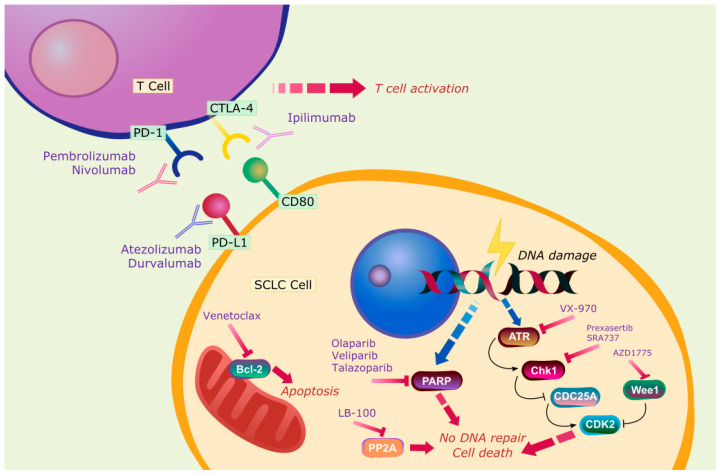
Current investigational immunotherapies and targeted therapies for small cell lung cancer SCLC.

Immunotherapy for SCLC was considered viable due to frequent somatic mutations as a result of smoking and the presence of paraneoplastic disorders [34,35,36]. Furthermore, in light of the remarkable success seen in NSCLC, parallel studies undertaken in SCLC have also shown considerable promise for immunotherapies that include antibodies against programmed cell death protein 1 (PD-1), programmed death-ligand 1 (PD-L1), and cytotoxic T-lymphocyte antigen 4 (CTLA4; Figure 1) [37,38] discussed below.

### 3.1. Atezolizumab

In treatment-naïve ES-SCLC patients, a recently published a phase III trial involving 403 patients, IMpower-133, combining atezolizumab with carboplatin and etoposide (EP) demonstrated an improved progression-free survival (PFS) as well as overall survival (OS) [39]. More specifically, the patients who did not progress after 4 cycles of induction therapy, received atezolizumab or placebo as maintenance every 3 weeks until disease progression or intolerable toxicity. Median OS for those treated with atezolizumab was 12.3 months compared to 10.3 months for the placebo group, with a hazard ratio for death of 0.70. Median PFS was also improved in the atezolizumab group, which was 5.2 months vs. 4.3 months, with a hazard ratio for disease progression at 0.77, resulting in the approval of atezolizumab with EP for ES-SCLC in the first-line setting. However, blood-based tumor mutational burden (TMB) was not associated with clinical benefit in this study.

### 3.2. Durvalumab

Another phase III trial, the CASPIAN trial, which used durvalumab as the immunotherapy in combination with platinum with etoposide to treat treatment-naïve ES-SCLC patients, also showed improvement in OS compared to platinum-etoposide alone (13 months vs. 10.3 months, with a hazard ratio of 0.73) [40]. Based on these results, the Food and Drug Administration (FDA) also approved durvalumab for ES-SCLC.

### 3.3. Ipilimumab and Nivolumab

In contrast to atezolizumab or durvalumab, ipilimumab (an anti-cytotoxic T-lymphocyte-associated protein 4 (CTLA4) antibody) in combination with chemotherapy prolongs PFS, but does not improve OS in treatment-naïve ES-SCLC [41]. However, maintenance therapy in such patients with nivolumab/ipilimumab combination or nivolumab alone did not show improvement in OS, according to results from the phase III CheckMate 451 study presented at the recent European Lung Cancer Congress 2019 [42]. Another trial CheckMate 032 assessed nivolumab as a single agent or in combination with ipilimumab in previously treated SCLC and found that ORR with single agent nivolumab was 11% compared to 22% in the cohort with combination of nivolumab with ipilimumab. The median OS for nivolumab alone was 4.1 months, and for nivolumab with ipilimumab, it was 6 months to 7.8 months based on the doses received [43]. Because the long-term survival benefits with nivolumab alone demonstrated better outcomes compared to previous agents used in the third-line setting, nivolumab received FDA approval for third-line treatment of SCLC.

### 3.4. Pembrolizumab

Pembrolizumab was studied in relapsed SCLC patients in the KEYNOTE-028 and KEYNOTE-158 trials. In KEYNOTE-028, the study included only patients with PD-L1 combined positive score (CPS) ≥1%. Among 24 patients with relapsed SCLC, 12.5% were treated with pembrolizumab in the second-line setting and 50% in the third-line. ORR was 33%, median PFS was 1.9 months, one-year PFS was 23.8%, median OS was 9.7 months, and the one-year OS was 37.7% [44]. In the KEYNOTE-158 trial, 79% of 107 patients with relapsed SCLC were treated with pembrolizumab in the second-line or third-line setting. A total of 47% of patients were PD-L1-negative, with an ORR of 18.7% (35.7% in the PD-L1-positive subgroup and 6.0% PD-L1-negative subgroup). The median PFS was 2 months, and median OS was 9.1 months [45]. This led to the approval of pembrolizumab in metastatic SCLC patients whose disease progresses on or after platinum-based chemotherapy and at least one other line of treatment. Considered together, although immunotherapy appears promising for SCLC patients, its benefits are modest, and there is significant room for further improvement.

## 4. Targeted Therapy

Unlike in the case of NSCLC, there are currently no targeted therapies available for SCLC. The lack of knowledge of the key genetic mutations and molecular targets that drive SCLC initiation and progress to a more aggressive disease has been a major impediment in developing targeted therapies. However, recent genome-wide studies have identified the universal loss of tumor suppressor genes such as tumor protein 53 (TP53) in 75–90% of patients and retinoblastoma 1 (RB1) and by frequent 3P deletion [46,47,48,49,50,51]. Consistent with these observations, studies using genetically engineered mouse models have confirmed that the introduction of these two events in pulmonary cells can give rise to high frequency of SCLC development [52]. Nonetheless, more than 120 clinical trials are ongoing that are evaluating new drugs in SCLC targeting various/multiple pathways. Below, we review a few key studies and are depicted in Figure 1.

Aberrant signaling driven by epidermal growth factor receptor (EGFR), stem cell factor receptor tyrosine kinase (c-KIT), PI3K/AKT/mTOR, insulin-like growth factor receptor (IGFR1), and hedgehog signaling pathways have been identified in SCLC. However, inhibitors targeting these pathways have shown minimal efficacy in first-line, maintenance and relapsed SCLC [34,53,54,55,56,57,58,59,60,61,62,63,64,65,66,67,68,69,70]. Additionally, overexpression and amplification of MET and fibroblast growth factor receptor (FGFR) that are associated with regulating cell proliferation, survival, motility, ability of invasion, and chemoresistance were also observed in SCLC. However, therapies targeting these pathways have not fared well and next generation inhibitors need to be evaluated in combination with either chemotherapy or immunotherapy [71,72,73,74,75,76].

The apoptotic pathway and the cell cycle checkpoint are some of the other pathways that have been targeted in SCLC. In the former case, B-cell lymphoma 2 (BCL-2) is the favorite therapeutic target. However, BCL-2 antisense oligonucleotide oblimersen and other agents, including obatoclax and navitoclax, have not shown significant activity against SCLC in both phase I and phase II trials [77,78]. Another BCL-2-specific inhibitor venetoclax, demonstrated efficacy in SCLC cell lines expressing high levels of BCL-2 [79] and a phase I trial of venetoclax together with ABBV-075, a bromodomain and extra-terminal domain (BET) inhibitor, is currently under way (NCT02391480). As far as the cell cycle checkpoint is concerned, ataxia telangiectasia and Rad3-related protein (ATR), checkpoint kinase-1 (CHK1), WEE1, and aurora kinase (AURKA), have been the preferred targets among others [80].

In addition to the pathways discussed above, transcription and DNA repair pathways have also been investigated in SCLC. Of these, the MYC pathway stands out since MYC is amplified in a significant (~20%) fraction of SCLC patients and appears to have higher sensitivity to certain newer targeted therapies, such as AURKA and BET inhibitors [81,82,83,84]. Other agents that are currently being evaluated are: chiauranib, an aurora B kinase inhibitor for relapsed SCLC (NCT03216343), GSK525762, a BET inhibitor as monotherapy for patients harboring MYC amplification (NCT01587703), and in combination with trametinib for patients carrying RAS mutations (NCT03266159).

Wee-like protein kinase 1 (WEE1) is a key tyrosine kinase involved in halting the G2-to-M phase transition of the cell cycle upon DNA damage [85] that is overexpressed in SCLC [86]. In a preclinical study, the combination of Poly(ADP-Ribose) Polymerase (PARP) inhibitors and WEE1 inhibitors demonstrated a synergistic effect [87]. A phase II, multi-arm trial (BALTIC) is currently evaluating the efficacy of novel therapies in patients with ES-SCLC refractory to platinum-based agents. These novel therapies include PD-L1 inhibitor durvalumab, PARP inhibitor olaparib, and WEE1 kinase inhibitor AZD1775.

Poly (ADP-ribose) polymerase 1 (PARP1) another key player in DNA repair is overexpressed in SCLC [88]. PARP inhibitors prevent cancer cells from repairing DNA damage caused by cytotoxic drugs. Several PARP inhibitors have demonstrated antitumor efficacy in preclinical SCLC models and are currently being studied in several clinical trials. A phase II study investigating veliparib in combination with cisplatin and etoposide in untreated ES-SCLC patients showed improvement in the primary endpoint PFS (6.1 months vs. 5.5 months) although no significant differences in OS were observed [89]. However, Schlafen-11 (SLFN11), which is involved in regulating response to DNA damage and is overexpressed in about 48% of SCLC, has been identified as a potential biomarker for veliparib benefit [90]. Recently, another PARP inhibitor talazoparib also caused higher sensitization to radiotherapy in SCLC cell lines and patient-derived xenografts. Thus, PARP inhibitors have great potential to emerge as a promising therapy for SCLC [91].

Activation of the Notch pathway is oncogenic in some cancer types, but in SCLC, the inhibition of Notch pathway is involved in tumorigenic signaling, progression, and chemoresistance [92]. Consistently, the inhibitory Notch ligand Delta-like protein 3 (DLL3) is upregulated in 85% of SCLCs compared to normal lung [93]. Rovalpituzumab tesirine (Rova-T), a first-in-class DLL3 antibody-drug conjugate, initially exhibited promising results of 18% ORR in heavily pretreated SCLC [94]. Unfortunately, high toxicity rates in the phase II TRINITY trial (NCT02674568) precluded the study from meeting its primary endpoint. In addition, the phase III MERU trial (NCT03033511) evaluating Rova-T in the maintenance setting following first-line chemotherapy, was also terminated early as a result of lack of survival benefit at an interim analysis. Likewise, another phase III (TAHOE) study that assessed Rova-T as a second-line therapy for advanced SCLC compared to topotecan, stopped enrollment due to shorter OS in the Rova-T group compared with the topotecan control group [95,96]. A phase I/II study evaluating the safety of Rova-T administered in combination with nivolumab or nivolumab and ipilimumab for adults with ES-SCLC has been recently completed and could decide the future of Rova-T (NCT03026166).

## 5. Protein Phosphatase 2A (PP2A)

Protein phosphatase 2A (PP2A), a serine/threonine phosphatase that functions as a tumor suppressor in many cancers [97], is also involved in various cellular processes, such as protein synthesis, cellular signaling, cell cycle, apoptosis, metabolism, and stress responses [98]. Several small-molecule activators of PP2A (SMAPs) have emerged as first-in-class agents for this target [99,100,101,102]. Further, a recent study has shown that PP2A suppression leads to resistance to kinase inhibitors in KRAS-driven lung cancer cell lines. In KRAS-driven xenograft mouse models, combination treatment of SMAP and selumetinib (a MEK inhibitor used in clinical trials) led to significant tumor regression compared to either agent alone [103,104]. Although PP2A is generally held to have tumor suppressor function, several lines of evidence suggest that it could also function as an oncogene. Thus, small molecule inhibitors of PP2A such as LB-100 [105], are emerging as novel strategies for SCLC. Furthermore, since PP2A is also associated with immune response by downregulating cytotoxic T-lymphocyte function [106,107], PP2A inhibition combined with immunotherapy appears to be effective in mediating an antitumor response. Indeed, in colon cancer and melanoma cells, a combination of LB-100 and an immune checkpoint inhibitor led to greater T-cell-dependent anti-tumor response, with more effector T-cell and reduced regulatory T-cell infiltration [106]. Carbonic anhydrase IX (CAIX), an enzyme involved in hypoxia inducible factor 1 alpha (HIF-1α) hypoxic signaling, is a promising target for chimeric antigen receptor-T (CAR-T) cells in an intracranial mouse model for glioblastoma [108]. In this mouse model, LB-100 was shown to augment the cytotoxic response of anti-carbonic anhydrase (CAIX) CAR-T cells, underscoring the therapeutic potential of synergistic LB-100 and CAR-T cell therapy in SCLC and other solid tumors [109].

## 6. Mitochondria

Mitochondria play an essential role in cell survival, apoptosis, adenosine triphosphate (ATP) production, as well as tumorigenesis [110]. Multiple therapeutic strategies have been developed to target mitochondrial functions, such as oxidative phosphorylation, glycolysis, tricarboxylic acid (TCA) cycle, apoptosis, reactive oxygen species (ROS) regulation, permeability transition pore complex, mitochondrial DNA, and dihydroorotate de-hydrogenase (DHODH)-linked pyrimidine synthesis [111]. Drugs that target mitochondrial metabolism through inhibition of pyruvate dehydrogenase (CPI-613, dichloroacetate), isocitrate dehydrogenase (AG-22, AG-120, AG-881) and targeting apoptotic pathways (birinapant, Minnelide, ME-334, Debio 1143, ONC201, LCL161) have been studied in early phase clinical trials [112,113,114,115,116,117]. These drugs have modest clinical activity as single agents in various tumors including SCLC and are currently being explored as combination therapies with chemotherapy, immunotherapy, and other targeted therapies [118].

## 7. Stem Cell Therapy

Cancer stem cells (CSCs) are defined as a small population of cells within a heterogeneous tumor that exhibit similar traits of normal stem cells. CSCs can originate from either somatic stem cells or differentiated progenitor cells. They prompt tumorigenic activity by undergoing self-renewal and differentiation, leading to tumor relapse, resistance to therapy, and metastasis [119,120,121]. Hence, targeting CSCs has become a novel therapeutic strategy for cancer treatment. CSCs often have upregulated signaling involved in development and tissue homeostasis, such as Notch, Hedgehog and wingless type 1 (WNT) pathways, all of which can be found in SCLC [122,123]. Unfortunately, Notch signaling inhibition with Rova-T and tarextumab have failed, but combination therapies studies are ongoing that could potentially yield positive results. Lysine demethylase 1 (LSD1) is implicated in maintaining stemness properties and hence, has emerged as a potential target for inhibiting lung CSCs [124]. A phase II trial (CLEPSIDRA) investigating the LSD1 inhibitor iadademstat in combination with standard-of-care in relapsing SCLC patients showed remarkable response rates (up to 75%). Preclinical studies with another LSD1 inhibitor GSK2879552 using 150 cancer cell lines, showed that SCLC and acute myeloid leukemia (AML) cell lines were sensitive to growth inhibition by the LSD1 inhibitor [125,126,127]. Furthermore, dual inhibition of LSD1 and PD-1 appear to be more effective than either therapy alone [128]. Another route to target stem cells is through CD47 inhibition by RRx-001, which targets tumor-associated macrophages and CSCs via downregulation of the antiphagocytic CD47/SIRPα checkpoint axis. A phase II trial (QUADRUPLE THREAT) involving 26 previously platinum-treated third-line SCLC patients showed that the OS and PFS for patients treated with RRx-001 and a reintroduced platinum doublet were 8.6 months and 7.5 months, respectively, which is much higher for a third-line treatment reported in literature [129]. Interestingly, biopsies taken from patients have correlated response to CD47 inhibition with a high density of infiltrated tumor-associated macrophages that are abundant in SCLC. Based on these observations, a phase III (REPLATINUM) randomized study of RRx-001 with platinum doublet vs. only platinum doublet in third-line SCLC is currently ongoing (NCT03699956).

## 8. Improving Outcomes

### 8.1. Biomarker-Based Therapy

Although immunotherapy has improved median survival for treatment-naïve ES-SCLC patients, the benefits have been limited with improvement in both PFS and OS approximately 1 and 2 months, respectively, with only 12.6% of patients remaining progression-free after one-year [39]. To improve the outcomes, better selection of patients based on predictive biomarkers, given the high mutational load and rapid resistance in SCLC, and therapies targeting multiple pathways with combination strategies need to be developed.

PD-L1 remains the most common immune-based biomarker for several malignancies. PD-L1 staining in SCLC is less intense and infrequent compared to NSCLC [130]. In KEYNOTE-158, a combined score >1 for PD-L1 expression by the Dako 22C3 assay appeared to predict increased response to pembrolizumab and improved survival when compared with patients negative for PD-L1 [44]; however, the PD-L1 positivity based on Dako 28-8 assay as in the CheckMate 032 did not replicate those results [131]. The assays differ in that KEYNOTE-158 used a PD-L1 score based on staining of tumor cells, lymphocytes and macrophages, whereas CheckMate 032 used staining of only tumor cells to determine positivity. The ongoing phase II REACTION (NCT02580994) and the phase III KEYNOTE-604 (NCT03066778) trials will require measurement of PD-L1 at baseline to provide insight into the predictive role of PD-L1 expression. Higher tumor mutation burden (TMB) has been recognized as a likely predictor of response to immunotherapy across disease types [132]. In an exome-sequencing analysis of CheckMate 032, patients with high TMB appeared to have a greater improvement in OS when treated with nivolumab. Patients with high-, medium-, and low-TMB had a median OS of 5.4 months, 3.9 months, and 3.1 months, respectively; the one-year OS rates were 35.2%, 26.0%, and 22.1%, respectively [133]. However, in the IMpower-133, a blood-based TMB failed to predict benefit for atezolizumab, thus requiring further prospective randomized validation TMB studies. Circulating tumor cells (CTCs) can be detected in 85% of SCLC patients and can potentially serve as a biomarker [134]. CTCs have been explored in multiple studies as a biomarker to predict response and resistance to therapy; however, additional studies looking into the genomic, epigenetic, and transcriptomic heterogeneity of CTCs at diagnosis and during relapse need to be done before it can be applied in clinics [135,136]. Cell-free DNA (cfDNA) widely used in NSCLC has also been examined in SCLC. A study with 27 patients showed cfDNA was able to mirror treatment response and even identified disease recurrence before radiological progression [137]. Future work based on tumor or blood-based biomarkers will help a long way in understanding treatment resistance in SCLC.

### 8.2. Combination Therapy

To overcome treatment resistance, novel combination approaches targeting multiple pathways are being explored in combination with chemotherapy and immunotherapies. Lurbinectedin, a novel cytotoxic drug, is a transcriptional inhibitor that inhibits RNA polymerase II. In a phase II trial for both sensitive and resistant disease, lurbinectedin was active as a single agent in second-line SCLC with an ORR of 35.2% [138]. Based on this study, the FDA has approved lurbinectedin for the treatment of ES-SCLC patients with disease progression after platinum-based chemotherapy. A phase III study (ATLANTIS trial, NCT02566993) of lurbinectedin in combination with doxorubicin vs. chemotherapy has completed recruitment and results are pending. Current trials investigating targeted therapies with or without chemotherapy include WEE1 inhibitor AZD1775 in combination with carboplatin (NCT02937818) and olaparib (NCT02511795), checkpoint kinase 1 (CHK1) inhibitor SRA737 in combination with cisplatin/gemcitabine (NCT027979770), ataxia–telangiectasia and Rad3 related (ATR) inhibitor AZD6738 in combination with olaparib (NCT02937818), another ATR inhibitor VX-970 in combination with topotecan (NCT02487095), Bcl-2 inhibitor navitoclax and mTOR inhibitor vistusertib (NCT03366103), Bcl-2 inhibitor venetoclax and ABBV-075 (NCT02391480), and Aurora B kinase inhibitor (NCT02579226) are all ongoing, which should shed some light on the future of targeted therapy in SCLC. There are numerous early phase trials investigating the combination of immunotherapy and targeted drugs as well. Durvalumab with olaparib (NCT02734004, MENDIOLA), avelumab with utomilumab, which is a humanized monoclonal antibody (mAb) that stimulates signaling through CD137 (NCT02554812), nivolumab plus ipilimumab with dendritic cell-based p53 vaccine 9 (NCT03406715) in relapsed SCLC, atezolizumab with chemotherapy and a CDK 4/6 inhibitor trilaciclib (NCT03041311) in first-line ES-SCLC are some of the novel combinations of immunotherapy with targeted agents.

### 8.3. Other Modalities

Other modalities of therapies targeting cell surface antigens expressed on tumor cells by monoclonal antibodies or surface-targeting immunotherapies, such as CAR-T cells and bispecific T-cell engagers (BiTEs), are in early stages of development. CD56 is expressed in almost all SCLC tumors, and thus, presents to be an attractive target for treating SCLC [139]. In a preclinical study, promiximab-duocarmycin (DUBA), a CD56 antibody conjugated to a potent DNA alkylating agent with a novel linker, showed promising results. This antibody drug conjugate (ADC) demonstrated high efficacy in SCLC xenograft models [140], suggesting that further clinical evaluation of this compound may be beneficial. Another ADC sacituzumab govitecan is comprised of a humanized mAb targeting Trop-2 (trophoblastic antigen-2), which is highly expressed in several epithelial cancers [141], fused to SN-38 (the active metabolite of irinotecan), which induces double- and single-strand DNA breaks by inhibiting topoisomerase I [142]. Sacituzumab govitecan is currently being investigated in several trials, including a phase I/II trial where it is being evaluated as a single agent in patients with previously treated, advanced SCLC (NCT01631552). CAR-T cells targeting DLL3 have entered a phase I clinical trial (NCT03392064). AMG 757, a BiTE, is also being evaluated in a phase I trial that includes ES-SCLC patients requiring first-line maintenance therapy and those with SCLC recurrence (NCT03319940). In patients with metastatic solid tumors including relapsed SCLC, targeting other immune checkpoints, such as PD-1 and CTLA-4, with immunotherapies, including TIM3 and LAG3, are being evaluated in clinical trials in combination with anti-PD-1 or anti-PD-L1 antibodies (NCT03708328, NCT03365791). Finally, radiation therapy is assumed to modulate immune response, as it can increase tumor antigen production and presentation and also enhance cytotoxic T-lymphocyte activity [143]. Potential synergy of radiotherapy in combination with immunotherapy in patients with ES-SCLC is being evaluated in innovative ongoing trials, and results are expected in the near future. Altogether, advancement in biomarkers, targeting multiple critical pathways, and enhancing immunotherapy efficacy in SCLC will hopefully improve the survival outcomes for SCLC patients, which has been elusive for many years.

## 9. Community Network-City of Hope Experience

**Figure 2:**
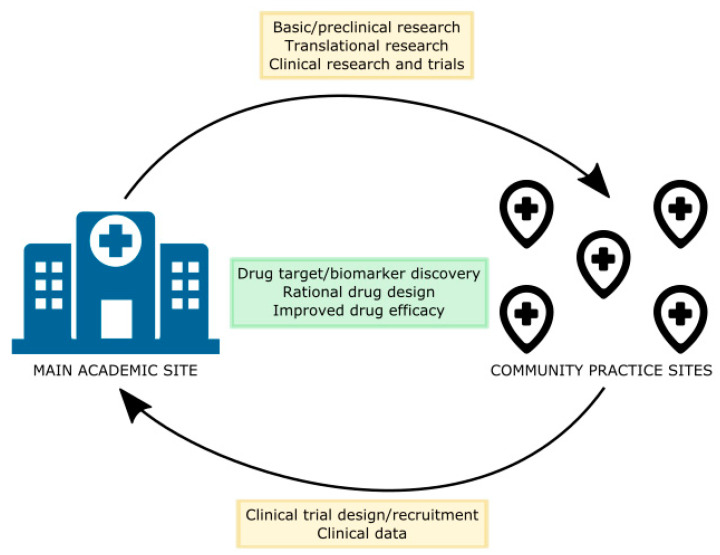
Collaboration between main academic site and community sites for clinical research.

City of Hope is a National Cancer Institute (NCI)-designated Comprehensive Cancer Center and a member of the National Comprehensive Cancer Network (NCCN). In addition, all clinical sites accept the Via Oncology Pathways (modified by City of Hope) for evaluation, antitumor treatment and surveillance after treatments have concluded. City of Hope is composed of a central academic site in Duarte and several satellite sites within Southern California. At the academic center, preclinical work is performed, and clinical trials on that translational research can be rapidly deployed across the entire enterprise, making bench-to-bedside research feasible and fascicle. The collaboration between basic research done on the main campus and clinical research done at both the main and satellite campuses, furthers the discovery of disease biomarkers and novel drug targets. This results in more rational drug design, improved therapeutic efficacy, and quicker optimization of high priority drugs for clinical use (Figure 2).

Clinical trials are initiated at the academic campus in Duarte and at one or more of our 27 affiliated network community cancer center offices that are staffed with 43 medical oncologists, 40 radiation oncologists, seven advanced practice providers (APPs) and a clinical trials coordinator. As directed by the Recalcitrant Cancer Research Congressional Act of 2012 (H.R.733), the National Cancer Institute (NCI) allocates resources for research and treatment of recalcitrant cancers having five-year relative survival rates of <20% and estimated to cause at least 30,000 deaths in the US per year. SCLC is considered a recalcitrant cancer having a dismal five-year survival rate of less than 7%. One of the major limitations to ongoing research in SCLC is tumor tissue availability, as the disease is rarely treated surgically. Another issue is many clinical trials in SCLC cannot be completed due to lack of accrual. Using the academic collaboration model with the academic center along with 27 community sites, enrollment becomes more feasible. The rapid progression of disease in SCLC relapse also places research on an urgent timeline to test new agents with a small window to observe treatment efficacy. Given single Institutional Review Board (IRB) approval in our institution, clinical trials can be opened at multiple sites in a rapid fashion. At the academic site, preclinical investigations are done, and clinical trials based on translational research data can be rapidly designed and disseminated across the entire enterprise, facilitating bench-to-bedside SCLC research in a more feasible manner. The collective knowledge gained from the interaction between the academic and community sites will provide insight into how to overcome challenges that continuously hinder therapeutic advancements in SCLC.

## 10. Future Directions

Traditionally, SCLC has been regarded as a homogenous disease, which led to most SCLC patients being treated with essentially one standard regimen. Recent studies from molecular analysis of patient tissues and genetically defined models indicate that there is notable heterogeneity among SCLCs in terms of histology, growth characteristics, expression of neuroendocrine cell differentiation markers, MYC activation, Notch pathway inactivation, and role of neuronal lineage-specific transcription factors in this disease (ASCL1, achaete-scute homologue 1; NeuroD1, neurogenic differentiation factor 1; POU2F3, POU class 2 homeobox 3; YAP1, yes-associated protein 1) [144,145,146,147,148]. Currently, SCLC is classified into four subtypes based on increased expression of different markers: ASCL1 high (SCLC-A), NEUROD1 high (SCLC-N), POU2F3 high (SCLC-P), and YAP1 high (SCLC-Y) [79]. SCLC-A and SCLC-N are neuroendocrine subtypes, whereas SCLC-P and SCLC-Y are non-neuroendocrine subtypes. These subtypes can be associated with specific biomarkers that are either drug-specific targets or predictors of drug response (e.g., DLL3 in SCLC-A, AURKA in SCLC-N, CDK4/6 in SCLC-Y and IGF1R in SCLC-P). These distinct gene expression profiles will guide us in designing new clinical trials. Recent advances in using patient-derived xenograft (PDX) models based on biopsy/resected tumors, CTCs, genetically engineered mouse models (GEMM), as well as omics profiling will drastically enhance our capacity to identify and test novel drugs and discover biomarkers for treatment and prognostication [93,149].

In conclusion, we recommend: (i) setting up a centralized biobank and repository leading to creation of a database incorporating full genomic, proteomic, and microRNA information; (ii) enrolling a higher proportion of SCLC patients into clinical trials with obligatory biomarker analysis; (iii) creating a master protocol which will help reduce duplicative effort and thus ease the eligibility requirements for clinical trials; (iv) create and incentivize academic and community research partnership centers of excellence, since most SCLC patients are treated in community sites; (v) collaborate with bioengineers, cancer biologists, and biophysicists to gather the genetic aberrations discovered and harness the power of computational modeling of genetic information, which will be a powerful tool in understanding SCLC and developing future therapies. Given the academic and community partnership we have established at City of Hope, this should be achievable and pave way for success in treating this challenging disease.
